# P07 Hospital-acquired infections post admission in the vascular surgery department: epidemiology, clinical impact and 30 day mortality in North Wales, United Kingdom

**DOI:** 10.1093/jacamr/dlaf230.014

**Published:** 2025-12-04

**Authors:** Armia Sargious, Faisal M Shaikh, Muhammad Mohsin, Buddug Ecklay, Abigail Williams, Sudhara Budagoda, Mohan Devbhandari, Laszlo Papp

**Affiliations:** Vascular Surgery Department, North Wales, Betsi Cadwaladr University Health Board, UK; Vascular Surgery Department, North Wales, Betsi Cadwaladr University Health Board, UK; Vascular Surgery Department, North Wales, Betsi Cadwaladr University Health Board, UK; Vascular Surgery Department, North Wales, Betsi Cadwaladr University Health Board, UK; Vascular Surgery Department, North Wales, Betsi Cadwaladr University Health Board, UK; Vascular Surgery Department, North Wales, Betsi Cadwaladr University Health Board, UK; Vascular Surgery Department, North Wales, Betsi Cadwaladr University Health Board, UK; Vascular Surgery Department, North Wales, Betsi Cadwaladr University Health Board, UK

## Abstract

**Background:**

Hospital-acquired infections (HAIs) are a significant concern in vascular surgery, contributing to increased morbidity, mortality and healthcare costs. This study will evaluate the incidence, patterns and clinical impact of HAIs among vascular inpatients over a 14 month period at a single vascular centre. The paper measures the frequency and distribution of various infection types, analysing associated length of stay and mortality. While extensive research exists on specific hospital-acquired infections such as ventilator-associated pneumonia and catheter-associated urinary tract infections, there is a notable scarcity in the literature comparing the incidence, distribution and clinical impact of all infection types within a single vascular surgery centre.

**Methods:**

We conducted a retrospective analysis of all patients admitted in vascular surgery, from 1 June 2020 to 31 July 2021 who developed new HAIs post-admission at our institute in North Wales (UK). Our centre is the vascular hub serving all the North Wales hospitals. Data were collected on demographics, procedure type, infection site, length of stay (LOS), mortality and re-intervention rates. Exclusion criteria included diabetic foot infections, pre-existing surgical site infections (SSIs) and *Clostridioides difficile* infection.

**Results:**

During the study period 943 patients were admitted in our department. A total of 157 patients (16.6%) developed new infections post admission, with male predominance (55%) and 85% occurring post emergency admissions. Infections were stratified into two groups. Chest infections and/ or urinary tract infections were more common (77%, 122/157) and classified as Group A: chest infections (55%, 67/122) or UTI (34%, 41/122) or both (11%, 14/122). Their mean LOS was 31 days and 30 day mortality was 29%. There were only 35 cases (22%) who presented with neither chest infections nor UTI. This group was classified as Group B and revealed bacteraemia and surgical site infections (SSI). Their mean LOS was 28 days; 30 day mortality was 20%. Classifying all infections into single versus combined infections showed that combined infections (45/157) had the longest average LOS (39 days). Isolated bacteraemia (4/157) showed the highest mortality (50%), followed by isolated chest infections (35%; 18/51) and combined infections (33.3%; 15/45). Mortality was lower in pure UTIs (13.3%; 4/30) and pure SSIs (11%; 3/27).

**Conclusions:**

Chest infections and UTI accounted for the majority of HAIs and were associated with prolonged hospital stays and nearly 30% mortality. Although less frequent, bacteraemia carried the highest mortality, underscoring its clinical severity. Combined infections significantly increased LOS and were associated with high mortality rates. These findings highlight the need for targeted infection prevention strategies and early intervention protocols in vascular surgical care to reduce the burden of HAIs and improve patient outcomes.

